# Risk factors associated with mortality in ıntensive care COVID-19 patients: the importance of chest CT score and intubation timing as risk factors

**DOI:** 10.3906/sag-2101-89

**Published:** 2021-08-30

**Authors:** Vecihe BAYRAK, Nurcan ŞENTÜRK DURUKAN, Ferhan DEMİRER AYDEMİR, Begüm ERGAN, N. Sinem GEZER, Oya Özlem EREN KUTSOYLU, A. Necati GÖKMEN, Yusuf SAVRAN

**Affiliations:** 1 Department of Internal Medicine, Intensive Care Unit, Faculty of Medicine, Dokuz Eylül University, İzmir Turkey; 2 Department of Public Health, Faculty of Medicine, Dokuz Eylül University, İzmir Turkey; 3 Department of Chest Diseases, Intensive Care Unit, Faculty of Medicine, Dokuz Eylül University, İzmir Turkey; 4 Department of Radiology, Faculty of Medicine, Dokuz Eylül University, İzmir Turkey; 5 Department of Infectious Diseases, Faculty of Medicine, Dokuz Eylül University, İzmir Turkey; 6 Department of Anesthesiology and reanimation, Intensive Care Unit, Faculty of Medicine, Dokuz Eylül University, İzmir Turkey; 7 Department of Internal Medicine, International Medicana Hospital, İzmir Turkey

**Keywords:** COVID-19, ICU mortality, CT score, intubation timing, APACHE II

## Abstract

**Background/aim:**

Coronavirus disease 2019 (COVID-19) is a disease with a high rate of progression to critical illness. However, the predictors of mortality in critically ill patients admitted to the intensive care unit (ICU) are not yet well understood. In this study, we aimed to investigate the risk factors associated with ICU mortality in our hospital.

**Materials and methods:**

In this single-centered retrospective study, we enrolled 86 critically ill adult patients with COVID-19 admitted to ICU of Dokuz Eylül UniversityHospital (İzmir, Turkey) between 18 March 2020 and 31 October 2020. Data on demographic information, preexisting comorbidities, treatments, the laboratory findings at ICU admission, and clinical outcomes were collected. The chest computerized tomography (CT) of the patients were evaluated specifically for COVID-19 and CT score was calculated. Data of the survivors and nonsurvivors were compared with survival analysis to identify risk factors of mortality in the ICU.

**Results:**

The mean age of the patients was 71.1 ± 14.1 years. The patients were predominantly male. The most common comorbidity in patients was hypertension. ICU mortality was 62.8%. Being over 60 years old, CT score > 15, acute physiology and chronic health evaluation (APACHE) II score ≥ 15, having dementia, treatment without favipiravir, base excess in blood gas analysis ≤ –2.0, WBC > 10,000/mm³, D-dimer > 1.6 µg/mL, troponin > 24 ng/L, Na ≥ 145 mmol/L were considered to link with ICU mortality according to Kaplan–Meier curves (log-rank test, p < 0.05). The APACHE II score (HR: 1.055, 95% CI: 1.021–1.090) and chest CT score (HR: 2.411, 95% CI:1.193–4.875) were associated with ICU mortality in the cox proportional-hazard regression model adjusted for age, dementia, favipiravir treatment and troponin. Howewer, no difference was found between survivors and nonsurvivors in terms of intubation timing.

**Conclusions:**

COVID-19 patients have a high ICU admission and mortality rate. Studies in the ICU are also crucial in this respect. In our study, we investigated the ICU mortality risk factors of COVID-19 patients. We determined a predictive mortality model consisting of APACHE II score and chest CT score. It was thought that this feasible and practical model would assist in making clinical decisions.

## 1. Introduction

In December 2019, the emergence of a novel coronavirus (2019-nCoV or severe acute respiratory syndrome coronavirus 2-SARS-CoV-2) in Wuhan, China’s Hubei province, triggered a pandemic [1]. As a result of the pandemic, the rates of hospitalization in an intensive care unit (ICU) are high in those with the coronavirus disease 2019 (COVID-19) that developed with SARS-CoV-2 [2].

In previous studies on COVID-19, the general characteristics of patients were defined [3,4]. The data required to reduce mortality in ICU patients are scarce. However, little attention has been paid to the clinical characteristics and prognosis of the ICU patients. More studies are certainly needed for risk factors of mortality in the ICU patients. Identifying these risk factors will help determine high-risk patients who may benefit from the close follow-up, aggressive supportive care, and early intervention. Imaging findings and the time from admission to the hospital to intubation will also help choose the patient group that will benefit from early intervention. In a few studies with a small number of patients, the effect of intubation timing on survival has been investigated, but exact results have not been obtained [5].

The chest CT score can determine the degree of virus-specific destruction in the lung parenchyma. Therefore, CT score was thought to determine disease severity more accurately than nonspecific inflammatory markers [6]. Based on this prediction, our study was planned that the CT score could be a good predictor of mortality. Many studies have investigated the relationship between score and disease severity [7,8]. However, a few studies have focused on score and prognosis. Several studies have investigated the effect of radiological evaluations at the time of admission on prognosis [9,10]. Our study was aimed to evaluate the CT score, intubation timing, and other risk factors for ICU mortality of the COVID-19 patient, and the results were analyzed.

## 2. Materials and methods

The retrospective observational study was performed with approval from the Dokuz Eylül University Ethics Committee (approval number 2020∕24−23) and the Turkish Ministry of Health (approval date 05/07/2020). The first 86 adult (> 17 years old) patients admitted to the adult intensive care unit of Dokuz Eylül University Hospital, between 18 March 2020 and 31 October 2020 were included in the study. The demographic characteristics (age, gender, comorbidities, COVID-19 diagnosis), acute physiology and chronic health evaluation (APACHE) score, laboratory and radiological findings were recorded retrospectively. The relationship of all these recorded data with ICU mortality was analyzed. 

### 2.1. Diagnosis 

COVID-19 was confirmed by positive reverse-transcriptase polymerase chain reaction (RT-PCR) and/or chest computerized tomography (CT) compatibility in all patients [11–13]. From the first day the patients were admitted to the hospital, all tests performed were taken into consideration. Respiratory samples were taken from the patient to identify the SARS-CoV-2 infection. These samples were obtained by nasal and pharyngeal swabs or tracheal secretion aspiration. “COVID-19 RT-qPCR Detection Kit” (Bio-speedy, Ankara, Turkey) was used to detect SARS-CoV-2 RNA in respiratory tract specimens.

The target site was the SARS-CoV-2 RNA-dependent RNA polymerase (RbRp) gene fragment. Samples with a cycle threshold (Ct) value of <40 were considered positive. The extraction process, “Viral Nucleic Acid Isolation Kit” (Bio-speedy, Ankara, Turkey) was performed by the manufacturer’s instructions. A 64-channel multidetector CT scanner (Brilliance, Philips Medical Systems) was used with an imaging protocol as follows: 120 kVp, 80 mA, slice thickness 1 mm, and high-spatial-frequency reconstruction algorithm (bone algorithm), without intravenous contrast medium.

### 2.2. Admission and intubation

Because of the possibility of ICU bed shortage, all ICU admissions were decided according to Turkish Ministry of Health COVID-19 Guidelines^1^https://covid19.saglik.gov.tr/TR-66301/covid-19-rehberi.html [accessed 07/01/2021].. This was as follows: patients with respiratory rate of ≥ 30/min, dyspnea and increased work of breathing, SpO_2_ < 90% or < 70 mmHg (in room air), oxygen requirement ≥ 5 L/min with a nasal cannula, lactate > 2 mmol/L, hypotension (systolic blood pressure (SBP) < 90 mmHg, > 40 mmHg drops from usual SBP, mean arterial pressure (MAP) < 65 mmHg), skin hypoperfusion sign, organ dysfunction such as confusion, kidney and liver test abnormalities, thrombocytopenia, elevated troponin level and arrhythmia. Patients meeting one of these criteria were evaluated for ICU admission. 

If the patient was not intubated when he was admitted to the ICU, he was followed up primarily with high-flow nasal oxygen (HFNO). Patients with respiratory distress and severe hypoxemia under oxygen therapy (tachypnea, increased respiratory depth, dyspnea, use of accessory respiratory muscles, paradoxical breathing, respiratory alkalosis) were intubated. The intubation decision was made by the specialist who followed the patient in the ICU.

A lung protective ventilation strategy was applied for patients with acute respiratory distress syndrome (ARDS) who require mechanical ventilation. Days from hospitalization to intubation were recorded.

### 2.3. Clinical, laboratory, and radiological data

Blood tests performed within the first 12 h of ICU admission (complete blood count, electrolytes, kidney and liver function tests, high sensitivity (hs) troponin, D-dimer, ferritin, C-reactive protein (CRP), coagulation tests, glucose, albumin, lactate dehydrogenase (LDH) values were collected. Arterial blood gas analysis data were obtained in the first hour of admission. Strong ion difference (SID) was calculated by subtracting the chlorine value from sodium value in each patient.

The APACHE II score for each patient was calculated by MDCalc^2^https://www.mdcalc.com/apache-ii-score. [accessed 07/01/2021]., and the corrected calcium level was also calculated by MDCalc^3^ https://www.mdcalc.com/calcium-correction-hypoalbuminemia. [accessed 07/01/2021]..

All scans were reviewed for CT diagnosis of COVID-19 associated pneumonia. Based on previous publication, a suspected SARS-CoV-2 pneumonia diagnosis was established considering the following chest CT patterns: usually multifocal, bilateral, and peripheral ground-glass opacity, crazy-paving and consolidation [14,15].

 CT scans were classified according to the Radiological Society of North America (RSNA) Expert Consensus Statement on Reporting Chest CT findings related to COVID-19 as follows: (1) negative for pneumonia, (2) typical appearance, (3) atypical appearance, and (4) indeterminate appearance [16]. 

A semiquantitative scoring system was used to quantitatively estimate the CT scans’ pulmonary involvement, which demonstrates a typical and indeterminate appearance for COVID-19 [17]. Each of the 5 lung lobes was visually scored on a scale of 0 to 5, with 0 indicating no involvement; 1, less than 5% involvement; 2, 5%–25% involvement; 3, 26%–49% involvement; 4, 50%–75% involvement; and 5, more than 75% involvement. The total CT score was the sum of the individual lobar scores and ranged from 0 (no involvement) to 25 (maximum involvement). Atypical CT scans were not scored since radiologic findings were not compatible with COVID-19. Image analysis was performed by a board-certificated radiologist with 15 years of experience in thoracic radiology. 

All treatment modalities (hydroxychloroquine - 800 mg on day 1, 400 mg from day 2 to day 5; favipiravir - 3200 mg on day 1, 1200 mg from day 2 to day 10 and antibiotics) were recorded. The treatment was mainly decided according to Turkish Ministry of Health COVID-19 Guidelines¹.

### 2.4 .Outcomes

The primary outcome of the study was mortality risk factors of COVID-19 critical care patients.

### 2.5. Statistical analysis

Patients’ properties were described with median and interquartile range (IQR) or mean ± SD for continuous variables. Categorical variables were presented with a number (n) and percentage (%). The normality hypothesis was tested. To determine basic differences between ICU survivors and nonsurvivors groups, for continuous variables Student’s t test or Mann–Whitney-U test (according to distrubition type) and for categorical variables χ2 test or Fisher’s exact test was used. In order to determine risk factors of COVID-19 mortality, survival analysis was done. Categories from continuous variables were obtained using as threshold the median or mean ± SD value of the overall sample for Kaplan–Meier. Firstly, Kaplan–Meier was performed by log-rank test for each variable. The variables which had the p value below 0.20 in the univariate analysis of the cox proportional risk regression model was put in to multivariate model to determine hazard ratios for the ICU mortality risk factors of COVID-19 patients. The relationship between the patients’ demographic, clinical, laboratory and radiological characteristics at the time of admission to the ICU and the ICU mortality was estimated. We tested the proportional hazard assumption, assessing interactions with survival time and examining Schoenfeld residual plots. The hazard ratio (HR) along with the 95% CI were reported. P-values < 0.05 were considered significant. Statistical analyses were performed with R software (Version 1.2.1335). 

## 3. Results

**Figure 1:**
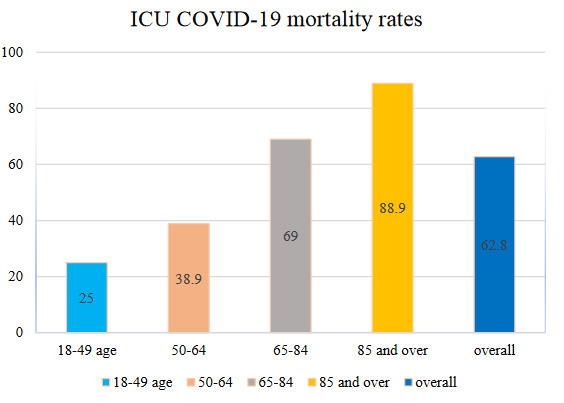
COVID-19 patients’ ICU mortality rate in different age groups.

Information of 86 inpatients in Dokuz Eylül University Hospital ICU, between 18 March 2020 and 31 October 2020 were collected. All patients were discharged or died before the date of data collection. Mean age of the patients was 71.1 ± 14.1 years, and predominantly male (70.9%) (Table 1). The overall mortality rate was 62.8%. The mortality rate of ICU in different age groups were shown in Figure 1. The median ICU survival was 12 days. Laboratory tests and other data obtained in ICU admission were compared between the survivor and nonsurvivor patients. All descriptive findings are shown in Tables 1–4. When comparing patients in ICU who nonsurvived and survived, the median age of nonsurvivors was 78.5 and survivors were 61.5 (p < 0.001) (Table1). In nonsurvivors, the median APACHE II score was higher than survivors (p < 0.05) (Table 2).

**Table 1:** Clinical and demographic characteristics of the patients.

Parameters	Overall(n = 86)	ICU survivors(n = 32)	ICU nonsurvivors(n = 54)	pvalues
Age (year), mean SD ±	71.1 ± 14.1	62.51 ± 12.5	76.2 ± 12.5	<0.001b b
18–49, n ( %)	8 (9.3)	6 (18.8)	2 (3.7)	
50–64, n (%)	18 (20.9)	11 (34.4)	7 (13.0)	0.002a
65–84, n (%)	42 (48.8)	13 (40.6)	29 (53.7)	
85 and over, n (%)	18 (20.9)	2 (6.3)	16 (29.6)	
Gender, n (%)				
Female	25 (29.1)	9 (28.1)	16 (29.6)	1.000a
Male	61 (70.9)	23 (71.9)	38 (70.4)	
Diagnosed by, n (%)				
PCR test	64 (74.4)	22 (68.8)	42 (77.8)	0.445a
Chest CT	22 (25.6)	10 (31.3)	12 (22.2)	
Admission from, n (%)				
Emergency service	16 (18.6)	5 (15.6)	11 (20.4)	0.776a
Pandemic service *	70 (81.4)	27 (84.4)	43 (79.6)	
Comorbidities, n ( %)				
Hypertension	54 (62.8)	19 (59.4)	35 (64.8)	0.650 a
Diabetes mellitus	31 (36.0)	13 (40.6)	18 (33.3)	0.643 a
CAD	19 (22.1)	4 (12.5)	15 (27.8)	0.115 a
COPD d disease	8 (9.3)	4 (12.5)	4 (7.4)	0.463 a
CHF	10 (11.6)	2 (6.3)	8 (14.8)	0.310 a
Dementia	10 (11.6)	0 (0.0)	10 (18.5)	0.011 a
CKD	8 (9.3)	2 (6.3)	6 (11.1)	0.704 a
Other diseases	18 (20.9)	4 (12.5)	14 (25.9)	0.176 a

a Chi-square test performe, b t test performed.

**Table 2:** Hospitalization, radiological characteristics, and outcomes of the patients.

Parameters	Overall (n = 86)	ICU survivors(n = 32)	ICU nonsurvivors (n = 54)	pvalues
Intubation before ICU, n (%) admission	36 (41.9)	5 (15.6)	31 (57.4)	<0.001a
Days from hospitalization to intubation, median(IQR)	3 (2.0–6.5)	4 (2–7)	3 (2–6)	0.739a
Not intubated patient, n (%)	15 (17.4)	15 (46.9)	0 (0.0)	<0.001c
Computerized tomography score (n: 71), mean ± SD	12.8 ± 4.7	11.6 ± 4.2	13.5 ± 4.8	0.090b
APACHE II score, median(IQR)	14.5 (9–20)	9 (8–13.5)	16 (14–22)	0.000a
Mortality day, median(IQR)	8.5 (3–14)	-	8.5 (3–14)	-
Length of ICU stay day, median (IQR)	8 (4–15)	7 (4.5–19)	8.5 (3–14)	0.736a

a Mann–Whitney U test performed, b t test performed, cchi-square test performed.

The mean of the CT score was found to be 12.8 ± 4.7. This value was 11.6 ± 4.2 in survivors and 13.5 ± 4.8 in nonsurvivors (Table 2). According to the patients’ CT evaluation results, 68 patients had a typical appearance, 12 patients had an atypical appearance, and 3 patients had an indeterminate appearance. Chest CT of three patients was taken outside the hospital before admission to ICU. These CT images were not included in the evaluation. It was thought that these would not comply with our standard.

**Figure 2:**
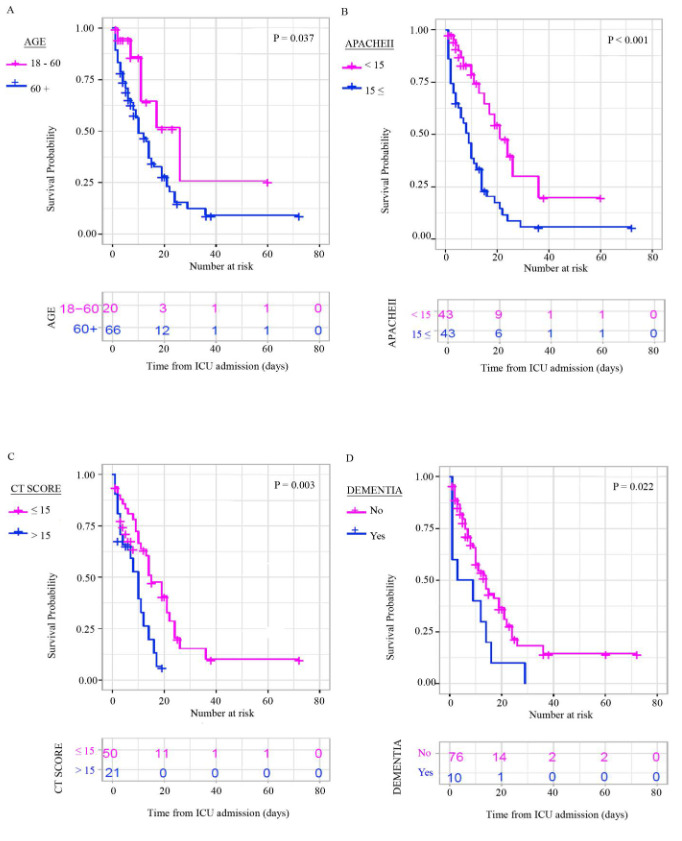

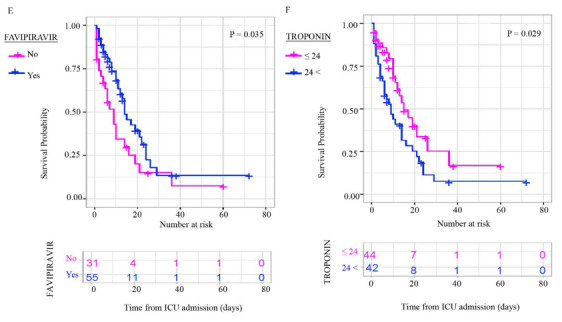
Kaplan–Meier survival plots for different prognostic factors.

Six of the Kaplan–Meier survival plots for the prognostic factors that resulted statistically significant are presented in Figure 2. Being over 60 years old, CT score > 15, APACHE II score ≥ 15, having dementia, treatment without favipiravir, base excess in blood gas analysis ≤ –2.0, WBC > 10,000/mm³, D-dimer > 1.6 µg/mL, troponin > 24 ng/L, and Na ≥ 145 mmol/L were considered to be linked with ICU mortality, according to Kaplan Meier (log-rank tests) performed on single risk factors (p < 0.05). The variables which had the p value below 0.20 in the univariate analysis of the cox proportional risk regression model was put in to multivariate model. A statistically significant model was done with CT score and APACHE II score (Table 5). The mortality risk was increased by 2.4 times in patients with a CT score ≥ 15 points than the patients with CT score < 15 points (HR: 2.411, CI 95%: 1.193–4.875) and the one point increase in APACHE II score increased the mortality risk by 5% (HR:1.055, Cl: 95%: 1.021–1.090). 

**Table 3:** Treatments of the patients at ICU admission.

Parameters		Overall (n = 86)	ICU survivors (n = 32)	ICU nonsurvivors (n = 54)	pavalues
Hydroxychloroquine, n (%)		41 (47.7)	11 (34.4)	30 (55.6)	0.075
Favipravir, n (%)		55 (64.0)	24 (75.0)	31 (57.4)	0.111
Antibacteriel agents, n (%)		51 (59.3)	21 (65.6)	30 (55.6)	0.376
Respiratory support, n (%)	Highflow nasal oxygen	50 (58.1)	27 (84.4)	23 (42.6)	<0.001
	Invasive mechanicalventilation	36 (41.9)	5 (15.6)	31 (57.4)	

aChi-square test performed.

**Table 4:** Blood gas analysis, biochemical parameters, and whole blood counts of the patients at ICU admission.

Parameters	Overall(n = 86) (n = 32)	ICU survivors(n = 32)	ICU nonsurvivors(n = 54)	p values
Blood gas analysis				
pH,median(IQR)	7.40 (7.30–7.40)	7.40 (7.30–7.40)	7.40 (7.20–7.40)	0.878a
PO2,mmHg, (UOT), median(IQR)	62 (56–83)	63 (59.5–87.5)	61 (55–82)	0.954a
O2 saturation, %, (UOT) ,median(IQR)	91.5 (88–96)	93.5 (90–96.5)	91 (86–95)	0.823a
PO2/FiO2 (UOT) ,median(IQR)	112.5 (101–154)	117.5 (109-156.5)	112 (96–154)	0.823a
PCO2, mmHg, median(IQR)	34 (30–43)	33 (30–38)	34 (30–45)	0.650a
HCO3, mmol/L, median(IQR)	22 (20–25)	24 (20.5–25.5)	21 (19-24)	0.114a
Lactate, mmol/L, median (IQR)	1.7 (1.2–2.4)	1.6 (1.1–2.1)	1.9 (1.3-2.6)	0.503a
Base excess, mmol/L,median(IQR)	–2.2 (–4.8–0.9)	0.0 (–3.0–1.8)	–3 (–5.0–0.4)	0.265a
Strong ion difference, median (IQR)	35 (33–38)	36.5 (35–38.5)	35 (33–37)	0.200a
Biochemical parameters				
Glucose, mg/dL, median (IQR)	146.5 (117–191)	134 (114–163.5)	150 (123-208)	0.265a
Sodium, mmol/L, median (IQR)	137.5 (133–141)	135.5 (131–140)	138 (134–142)	0.265a
Chloride, mmol/L, median (IQR)	101 (97–106)	99.5 (94.0–102.5)	102 (99–108)	0.177a
Calcium, mg/dL, median (IQR)	8.9 (8.6–9.2)	9.0 (8.6–9.2)	8.9 (8.6–9.2)	0.913a
Potasium, mmol/L, median (IQR)	4.2 (3.8–4.6)	4.3 (3.8–4.8)	4.1 (3.8–4.6)	0.341a
D-dimer, µg/mL, median (IQR)	1.6 (0.8–4.5)	1.1 (0.5–3.2)	2 (1–8)	0.014a
Ferritin, ng/mL, median(IQR)	613 (374–1063)	639 (425–1154.5)	551.5 (300–880)	0.265a
GFR, %, mean ± SD	63.7 ± 29.9	73.8 ± 28.9	57.7 ± 29.1	0.015b
Creatinine, mg/dL, median(IQR)	1.0 (0.8–1.5)	0.9 (0.8–1.3)	1.0 (0.7–1.8)	0.650a
ALT, U/L, median(IQR)	40.5 (23–68)	58 (35–89)	32 (19–51)	0.014a
LDH, U/L, median(IQR)	520 (398–655)			
Albumin, g/dL, median (IQR)	3.1 (2.8–3.3)	3.2 (2.9–3.4)	3.0 (2.6–3.2)	0.194a
Troponin, ng/L, median (IQR)	24 (8.4–102)	11.5 (6–28.5)	49.5 (11–128)	0.006a
CRP, mg/L, median (IQR)	150 (88–233)	148.5 (87.5–228)	158.5 (88v242)	0.823a
Whole blood counts				
Hemoglobin, g/dL, median (IQR)	12 (11–13)	12.5 (11–13.5)	12 (10–13)	0.341a
Hematocrit, %, mean ± SD	37.0 ± 5.7	38 ± 4.9	36.4 ± 6.0	0.185b
WBC,10³/UL, mean ± SD	10.2 ± 4.9	9.0 ± 3.5	11.0 ± 5.5	0.039b
Platelets, 10³/UL, median(IQR)	234.5 (167–301)	245.5 (167–294)	228.5 (167–294)	0.823a
Lymphocytes, /UL, median (IQR)	700 (500–1100)	700 (500–950)	800 (500–1200)	0.615a

aMann–Whitney U test performed, bt test performed.

**Table 5:** The model of CT score and APACHE II score.

Variables	Univariate analysis		Multivariate analysis (model)*	
	HR	CI (95%)	HR	CI (95%)
APACHE II	1.057	1.034–1.080	1.055	1.021–1.090
CT score (> 15)	2.519	1.308–4.850	2.411	1.193–4.875
Dementia	2.16	1.082–4.314		
Favipiravir	0.571	0.332–0.982		
Troponin	1.802	1.041–3.119		
Age	1.052	1.025–1.079		

*The model was adjusted for age, dementia, favipiravir, and troponin.

## 4. Discussion

In our study on COVID-19 patients in the ICU, the main findings included that APACHE II score, and chest CT score were independently associated with ICU mortality. No relationship could be shown between intubation timing and mortality. 

In the intensive care study data of COVID-19 patients, the mortality rate was high [18,19]. Results in our study were also consistent with these. It is thought that the high average age of our patients also affects this. In many studies, age has now been found a definite predictor of mortality for this disease [20,21]. Studies in patients with a critical illness, consistent with our study results, found higher mortality in elderly patients. 

A positive result was found in the univariate analysis of dementia comorbidity as a risk factor for ICU mortality. However, it did not effected the ICU mortality in multivariate model analysis. This result was obtained possibly due to factors such as age. Some studies have found that dementia may be a risk factor [22]. Studies included high number patients may research this issue. APACHE II prognostic score is widely used to predict mortality in ICU patients. In our study, initial APACHE II scores at admission were lower in survivor patients than nonsurvivor patients. This score was found significant in predicting ICU mortality also in multivariate analysis. 

The positive results of favipiravir use have been shown in many studies [23,24]. Treatment without favipiravir was seen as a risk factor for ICU mortality in the univariate analysis. However, in multivariate model analysis it was not significant. 

It was found that patients with critical COVID-19 were more likely to be intubated. Whether the timing of intubation was critical for a patient’s survival was investigated in some studies. Intubation timing was evaluated in a study in which 40 critical patients who had started high-flow oxygen and NIMV treatment were followed. It was found that survival was higher in patients who were intubated before 50 h, and the APACHE score was below 10 [5]. In another study, the effect of early and late intubation of 47 patients admitted to intensive care on mortality was investigated. There was no difference in mortality between those intubated on the day of ARDS and the next day [25]. In another study of 231 patients in intensive care, there was no difference in mortality between being intubated before the first 8 h, between 8 and 24 h, and after 24 h [26]. Similarly, the results of our study were that the timing of intubation was not associated with mortality. 

The clinical course of the COVID-19 is unpredictable due to the heterogeneity of its manifestations, ranging from asymptomatic forms to critical disease. There has been no currently available prognostic biomarker to identify patients requiring immediate medical attention and to estimate their associated mortality rate. In our study, we tried to find a marker by examining the factors affecting intensive care mortality. CT score was found to be significant in this respect. First, studies have been conducted to investigate the relationship between CT score and disease severity. Many studies have shown a positive relationship between this score and disease severity [27–29]. As a result, it was stated that detecting patients with severe disease by CT score would not provide sufficient information for these patients’ prognosis [7]. Therefore, as suggested, prognosis studies have started. In most of the studies examining the chest CT score as a marker, the patients’ prognosis from admission to the hospital was examined. Patients’ progression to severe disease was followed, the need for ICU was considered, but no exact data was found in terms of the relationship between chest CT score and ICU mortality. In two studies in which patients were evaluated using the chest CT scoring method, similar to our study, a correlation was found between the chest CT score and the increase in the patients’ oxygen need and the increase in disease severity [30]. Similar observations were reported by Colombi et al. [31], who found a positive correlation between the extent of CT lung involvement and ICU admission or death in a cohort of 236 patients. In another study, including 130 patients, it was found that a chest CT score above 18 was determinant for the short-term mortality of the patients [6]. In the study by Shuchang Zhou et al. [32], the CT score assessment was the same as in our study, and when the survivors and nonsurvivors were compared, the CT score above 16.5 showed a poor prognosis. However, the study was not conducted primarily with ICU patients. 

In our study, chest CT score above 15 was found to be associated with the mortality of COVID-19 ICU patients. Additionally, on a multivariable Cox proportional-hazard regression model APACHE II score and chest CT score were found independently associated with ICU mortality. Since this model includes clinical, laboratory and radiological parameters, it can help to evaluate the patient as a whole. The most important advantage of this model is that it is feasible and practical for most centers. In conclusion, CT scan and APACHE II score can have a pivotal role in assisting physicians in the management plan and work to indicate disease severity and possible outcome. Eventually, it may help to reduce the mortality rate of COVID-19.

Limitations of our study include its relatively small sample size and performance in a single medical center. 

In conclusion, we hope that the mortality data associated with COVID-19 from our study will assist in the early identification of individuals at risk of becoming critically ill and benefiting most from intensive care treatment. Further research on this disease, such as data collection and sharing and a critical review of the evidence will help the clinicians in clinical decision-making process.
